# Editorial: Come as you R(NA): post-transcriptional regulation will do the rest

**DOI:** 10.3389/fnmol.2025.1644067

**Published:** 2025-07-01

**Authors:** Oriane Mauger, Michael A. Kiebler, Clémence Bernard

**Affiliations:** ^1^Max Planck Institute of Psychiatry, Munich, Germany; ^2^Biomedical Center, Department of Cell Biology and Anatomy, Medical Faculty, LMU, Planegg, Germany; ^3^Department of Clinical and Biomedical Sciences, Faculty of Health and Life Sciences, University of Exeter, Exeter, United Kingdom

**Keywords:** RNA processing, neuron development and plasticity, translation regulation, neurological diseases, RNA-binding proteins

If RNA were simply a messenger between genes and proteins, cells would not function. RNA is a regulatory hub, a feature particularly leveraged in the central nervous system, where post-transcriptional processes (PTPs) control RNA stability, localization, translation and protein isoforms, mediating precise spatio-temporal control of gene expression (Alfonso-Gonzalez and Hilgers, 2024; Flamand et al., 2023; Ule and Blencowe, 2019). The extensive repertoire of PTPs, their widespread programs, the logic of their regulation and their physiological relevance have recently taken their full meaning. Indeed, PTPs tightly parallel the intricacy of the brain's spectacular diversity of cells with complex morphologies that need to integrate many extrinsic and intrinsic signals (Bauer et al., 2022, 2023; Darnell, 2013; Furlanis and Scheiffele, 2018; Holt et al., 2019). This editorial introduces the articles collected in this Research Topic to highlight the recent progress in the field of post-transcriptional control of gene expression in the central nervous system in health and disease (Figure 1).

**Figure 1:**
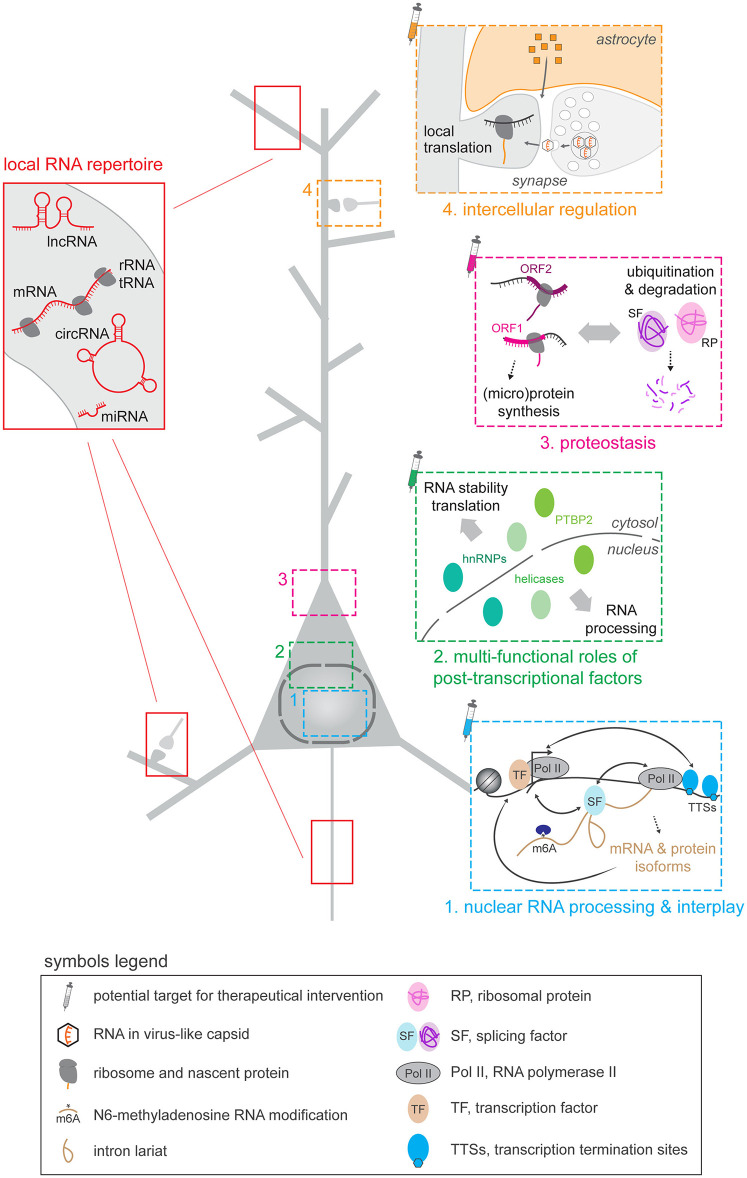
Post-transcriptional processes (PTPs) exert crucial spatio-temporal control of gene expression in the brain under basal conditions and in response to stimulation, a conserved feature observed across a wide range of species. These PTPs act upon a wide array of RNA categories in the various compartments formed by the complex morphology of neurons (**red box**). Within the nucleus **(1, blue box)**, various PTPs interact with each other and with other gene expression mechanisms, including transcription. The interplay of PTPs is further influenced by the dual localization of many PTP factors, which carry different functions in the nucleus and the cytosol to regulate RNA metabolism **(2, green box)**. Protein abundance is governed by tightly regulated protein synthesis, generating multiple protein and micro-protein forms, coupled with precise protein degradation **(3, pink box)**. The complex architecture of neurons requires local control of gene expression notably at synapses, involving the intercellular transfer of molecules such as astrocyte-secreted factors and RNA transported via virus-like capsids **(4, orange box)**. Many PTPs therefore represent potential targets for therapeutic interventions in a range of brain disorders.

## Diversity of post-transcriptional processes

While the diversity of PTPs has been known for several decades, new mechanisms are continuously revealed to have pivotal roles in shaping gene expression for brain development and function. For instance, epitranscriptomics represents a rapidly expanding area of research, with chemical modifications of mRNAs now emerging as being crucial for neurodevelopment and cognitive functions (Tegowski and Meyer). Dogmas in the PTP field are being revisited: for a long time, only one open-reading frame (ORF) per mRNA was thought to be active, ultimately giving rise to one protein isoform. Recent evidence has however revealed that multiple ORFs within the same mRNA can produce different protein isoforms. Many of these newly identified ORFs are often located in the improperly called “untranslated regions” of mRNA and code for microproteins that are likely relevant for neuronal cell functions (Duffy et al.). Beyond intracellular mechanisms, the intercellular transfer of secreted factors that influence PTPs (de la Cruz-Gambra and Baleriola) and of transcripts themselves (Taylor and Nikolaou) have started to be uncovered. For instance, factors secreted by astrocytes were found to regulate local translation of mRNAs located in neighboring neurons in culture (de la Cruz-Gambra and Baleriola). While PTPs are mainly investigated for coding RNAs, non-coding (nc) RNAs are also under post-transcriptional control. Many ncRNAs have been observed in neuronal processes including at synapses (Taylor and Nikolaou) with an increasing number shown to have coding capacity, revising our textbook vision of gene expression (Duffy et al.; Taylor and Nikolaou).

## Interplay between post-transcriptional mechanisms and other gene expression steps

The interplay between PTPs and other gene expression mechanisms is becoming increasingly evident and appears to control the availability, levels and isoforms of PTP factors. For instance, alternative splicing can control the production of transcription factor isoforms with distinct impact on neurodevelopmental transcription programs (Nazim). The expression levels of post-transcriptional factors can also be controlled by post-translational modifications. Ubiquitination—a key step of proteostasis—can target RNA-binding proteins (RBPs) such as splicing factors, and subsequently affect the splicing regime in the brain (Elu et al.).

This interplay is also clearly evidenced by the various functions exerted by PTP factors at different stages of the RNA life cycle. Many RBPs, such as RNA helicases and heterogeneous nuclear ribonucleoproteins (hnRNPs), exert distinct roles in different subcellular compartments (Lederbauer et al.; Tilliole et al.). The splicing factor poly-pyrimidine tract binding protein PTBP2 has also been shown to be transported in neuronal processes where it controls local translation (Salehi et al.). Finally, the interplay between PTPs and other gene expression processes can result from a local synergy, where RNA processing factors can be recruited at regulatory transcription regions such as promoters and enhancers. This crosstalk seems to play a pivotal role in dictating the developing neuronal transcriptome (Ozbulut and Hilgers).

## Specificity of post-transcriptional processes

Recent research has revealed a high specificity of PTPs, from subcellular localization to cell type and species differences. In neurons, specific PTPs are observed at the subcellular level, with neuronal processes and synapses exhibiting diverse molecular landscapes (Taylor and Nikolaou). More broadly, the many neuronal subtypes observed in the brain exhibit distinct transcripts and protein repertoires, to which different PTPs contribute. During development, cellular differentiation and specification are associated with dedicated PTPs (Ozbulut and Hilgers). Finally, another level of specificity is observed between species, raising the tantalizing hypothesis that PTPs also contribute to species divergence and precise features of individual species across evolution (Dando et al.).

## Post-transcriptional processes in disease

Several PTPs have been shown to be dysregulated in a range of neurological diseases. Pathological variants of RBPs such as RNA helicases and hnRNPs have been associated with neurodevelopmental disorders, including developmental delay, intellectual disability and brain anomalies (Lederbauer et al.; Tilliole et al.). Defects in RBPs have been linked to degenerative disorders, ranging from spinal muscular atrophy (Salehi et al.) to the frontotemporal lobar degeneration—amyotrophic lateral sclerosis spectrum and Alzheimer's disease (Tilliole et al.). Defective regulation of protein homeostasis has also been reported in several neurological disorders (Elu et al.).

A better understanding of PTPs in both health and disease opens the door to novel therapeutic means (Elu et al.; Salehi et al.). RNA-based tools such as splice-switching oligonucleotides have shown great promise to treat spinal muscular atrophy and amyotrophic lateral sclerosis (Zhang). RNA-targeting CRISPR-Cas9 technologies (Tegowski and Meyer) are also being developed, which will offer innovative options for therapeutical interventions.

## Technical challenges and looking forward

The recent progress described in the articles of this Research Topic is continuously accelerated by major technological advances, such as third generation sequencing and spatial transcriptomics (Taylor and Nikolaou). This is particularly exemplified by our recent ability to identify RNA modifications, and direct sequencing will provide more opportunities to study their effect at the single-molecule level (Tegowski and Meyer). More technical developments, such as single-synapse characterization and live imaging of translation, will bring unprecedented resolution to our understanding of the roles of PTPs in spatio-temporal control of gene expression. Artificial intelligence and machine learning will certainly revolutionize prediction of cis- and trans-regulatory elements. This will facilitate the implementation of emerging antisense oligonucleotide strategies to manipulate PTPs and investigate their functional relevance for neuronal circuits and cognition *in vivo*.

We hope that this Research Topic provides valuable material on the latest advances in PTP research and stimulates new avenues for our long-term goal to elucidate the foundational connections between these processes and brain function. The coming years will undoubtedly lead to a more precise understanding of the various levels of PTP regulation and their consequences, with impact on both basic science and translational investigations.

## References

[B1] Alfonso-Gonzalez C. Hilgers V. (2024). (Alternative) transcription start sites as regulators of RNA processing. Trends Cell. Biol. 34, 1018–1028. 10.1016/j.tcb.2024.02.010 38531762

[B2] Bauer K. E. Bargenda N. Schieweck R. Illig C. Segura I. Harner M.. (2022). RNA supply drives physiological granule assembly in neurons. Nat. Commun. 13:2781. 10.1038/s41467-022-30067-3 35589693 PMC9120520

[B3] Bauer K. E. de Queiroz B. R. Kiebler M. A. Besse F. (2023). RNA granules in neuronal plasticity and disease. Trends Neurosci. 46, 525–538. 10.1016/j.tins.2023.04.004 37202301

[B4] Darnell R. B. (2013). RNA protein interaction in neurons. Annu. Rev. Neurosci. 36, 243–270. 10.1146/annurev-neuro-062912-114322 23701460 PMC3889695

[B5] Flamand M. N. Tegowski M. Meyer K. D. (2023). The proteins of mRNA modification: writers, readers, and erasers. Annu. Rev. Biochem. 92, 145–173. 10.1146/annurev-biochem-052521-035330 37068770 PMC10443600

[B6] Furlanis E. Scheiffele P. (2018). Regulation of neuronal differentiation, function, and plasticity by alternative splicing. Annu. Rev. Cell Dev. Biol. 34, 451–469. 10.1146/annurev-cellbio-100617-062826 30028642 PMC6697533

[B7] Holt C. E. Martin K. C. Schuman E. M. (2019). Local translation in neurons: visualization and function. Nat. Struct. Mol. Biol. 26, 557–566. 10.1038/s41594-019-0263-5 31270476

[B8] Ule J. Blencowe B. J. (2019). Alternative splicing regulatory networks: functions, mechanisms, and evolution. Mol. Cell. 76, 329–345. 10.1016/j.molcel.2019.09.017 31626751

